# Feasibility and Safety of Early Post-COVID-19 High-Intensity Gait Training: A Pilot Study

**DOI:** 10.3390/jcm13010237

**Published:** 2023-12-31

**Authors:** Joakim Halvorsen, Christopher Henderson, Wendy Romney, Magnus Hågå, Tonje Barkenæs Eggen, Jan Egil Nordvik, Ingvild Rosseland, Jennifer Moore

**Affiliations:** 1 Forsterket Rehabilitering Aker, Helseetaten, Oslo kommune, Trondheimsveien 235, 0586 Oslo, Norway; magnus.haga@hel.oslo.kommune.no (M.H.); ingvildkristinahurum.rosseland@hel.oslo.kommune.no (I.R.); 2 Institute for Knowledge Translation, Carmel, IN 46033, USA; chende@knowledgetranslation.org (C.H.); jmoore@knowledgetranslation.org (J.M.); 3 Department of Physical Medicine and Rehabilitation, Indiana University School of Medicine, Indianapolis, IN 46254, USA; 4 Department of Physical Therapy and Human Movement Science, Sacred Heart University, Fairfield, CT 06825, USA; romneyw@sacredheart.edu; 5 Lom Kommune, Sognefjellsvegen 6, 2686 Lom, Norway; tonje.b.eggen@lom.kommune.no; 6 Faculty of Health Sciences, Oslo Metropolitan University, 0166 Oslo, Norway; janegiln@oslomet.no; 7 Regional Kompetansetjeneste for Rehabilitering, Sunnaas HF, Trondheimsveien 235, 0586 Oslo, Norway

**Keywords:** COVID-19, high-intensity gait training (HIT), feasibility, safety, subacute rehabilitation

## Abstract

Background: The feasibility and safety of rehabilitation interventions for individuals recovering from COVID-19 after the acute stage is not well understood. This pilot study aims to provide a preliminary investigation of the feasibility and safety of providing high-intensity gait training (HIT) with a targeted cardiovascular intensity of 70–85% of the age-predicted maximum heart rate (HRmax) for individuals undergoing rehabilitation post-COVID-19. Methods: Consecutive patients who were medically cleared for HIT were invited to participate in the study. Participants practiced walking in varied contexts (treadmill, overground, and stairs), aiming to spend as much time as possible within their target cardiovascular intensity zone during scheduled physical therapy (PT) sessions. Training characteristics and adverse events were collected to determine the feasibility and safety of HIT. The severity of adverse events was graded on a 1–5 scale according to the Common Terminology Criteria for Adverse Events. Results: The participants (*n* = 20) took a mean of 2093 (±619) steps per PT session. The average peak heart rate during PT sessions was 81.1% (±9.4) of HRmax, and 30.1% (±21.0) of the session time was spent at heart rates ≥ 70% HRmax. Mild adverse events (grade 1) occurred in <5% of the sessions, and no intervention-requiring or life-threatening adverse events (grade 2–5) occurred. Conclusion: This pilot study provides preliminary evidence that HIT may be feasible and safe during inpatient rehabilitation for patients post-COVID-19 following medical clearance.

## 1. Introduction

As of 27 August 2023, over 770 million confirmed cases of SARS-CoV-2 (COVID-19) leading to over 6.9 million deaths have been reported globally since the outbreak of the pandemic in 2019. Although the number of new cases is declining, over 1.4 million new cases and approximately 1800 deaths were reported between 31 July and 27 August 2023 [1]. Approximately 30% of patients who are hospitalized with COVID-19 require rehabilitation [2]. In addition, up to 40% of individuals who experienced severe COVID-19 continue to experience symptoms that may reduce function and quality of life one year after hospitalization (long COVID) [3].

Although the amount of research on COVID-19 is rapidly expanding, much of the research has focused on the prevention and acute management of the condition. The efficacy of rehabilitation interventions for individuals recovering from COVID-19 after the acute stage is not well understood. Early in the pandemic, Vitacca et al. (2020) suggested providing low-intensity exercise at cardiovascular intensities below three metabolic equivalents of task (METs) [4]. Interventions that maintain a cardiovascular intensity < 3 METs include light bodyweight resistance training and slow walking practice, while functional tasks such as sit-to-stand (squats), walking uphill, or stair climbing can increase the demand threefold [5,6,7]. Since inpatient rehabilitation aims to prepare the patients for the functional demands required to live in the home and community settings after discharge, avoiding these functional tasks could limit the interventions’ ability to meet a patient’s discharge needs.

More recent studies have identified potential benefits of exercising at higher cardiovascular intensities for individuals post-COVID-19. Exercise with high aerobic intensity increases the workload on the cardiovascular and pulmonary systems and may result in beneficial changes in health-related biomarkers for these systems [8,9,10]. Mohammed and Alawna (2020) theorized that increasing the aerobic capacity in people recovering from COVID-19 may lead to beneficial changes in immune cell function, lung elasticity and strength, and psychological conditions such as anxiety and depression [11]. In a systematic review, Alawna et al. (2020) analyzed the effects of aerobic exercise on immunological biomarkers and extrapolated results from other populations to recommend aerobic exercise between 60–80% of HRmax for 20–60 min 2–3 times per week in patients recovering from COVID-19 [12].

To date, only a few clinical studies have investigated the impact of training individuals who were recovering from COVID-19 at higher cardiovascular intensities. In three studies, participants post-COVID-19 have achieved intensities between 60–75% (i.e., moderate-intensity training [13]) of predicted HRmax [14,15,16]. In two studies on high-intensity interval training, the participants achieved intensities >85% during the intervals [17,18]. The results of these studies indicate that moderate-intensity training [14,15,16] and high-intensity interval training [17,18] are feasible post-COVID-19. Safety was investigated in one of the studies on moderate-intensity training [14] and in both studies on high-intensity interval training [17,18], with no severe adverse events reported in any of the studies. Outcomes following training at higher cardiovascular intensities include decreased severity and progression of COVID-19-associated disorders [15,18], improved physical [14,18] and psychological [16] function, and improved quality of life [15,16].

Although these results are promising, there are substantial methodological differences between the studies that affect their generalizability to an inpatient rehabilitation setting. Only two of the studies investigated participants with severe COVID-19 [14,17] and only three were performed in the acute or subacute stage after the disease [14,15,17]. Only the study by Mohamed and Alawna (2021) includes walking in the intervention description in addition to cycling [15]. Three of the studies investigated cycling training protocols [16,17,18], and one study investigated an arm ergometer protocol [14]. These activities can be argued to not be specifically functional during inpatient rehabilitation. Different definitions were also used for adverse events in the three studies that reported them [14,17,18].

To our knowledge, the feasibility and safety of routinely providing HIT, which focuses specifically on the provision of walking training while targeting high cardiovascular intensities, in an inpatient setting post-COVID-19 are unknown. This pilot study aims to provide a preliminary investigation of the feasibility and safety of HIT for this population by evaluating whether the participants can achieve a high number of steps and high cardiovascular intensities during inpatient PT sessions, without experiencing more frequent or severe adverse events than previously reported.

## 2. Materials and Methods

### 2.1. Study Sample and Design

The pilot study was conducted at Forsterket Rehabilitering Aker (FRA), Helseetaten, a rehabilitation unit in the primary healthcare system in the Oslo municipality, Norway. Adults aged >18 years old, who previously tested positive and were hospitalized for COVID-19, who were non-contagious, who underwent inpatient rehabilitation at FRA for functional deficits resulting from COVID-19, and who consented for their data to be utilized were included. The participants were also medically cleared by their supervising physician for participation in HIT and had no bracing or other instrumentation (e.g., ventilator-dependent) that substantially restricted movement.

### 2.2. Intervention

Patients were scheduled for physical therapy 60 min per day, five times per week (weekdays only). HIT was provided by the physical therapists four days per week, with one day set aside for administering outcome measures. As the pilot study was conducted in clinical practice, the number of HIT sessions was not predetermined as each participant’s length of stay (LOS) was based on an interdisciplinary evaluation of their rehabilitation needs.

The HIT protocol has previously been studied in individuals following stroke, spinal cord injuries, and those who are deemed medically frail [19,20,21,22,23] and is detailed in the appendix of Holleran et al. 2014 [19]. Some local adaptations were made to the targeted intensity, after discussions with the treating physician. Briefly, the protocol consists of maximizing the amount of stepping practice provided in variable contexts (e.g., treadmill, overground, and stairs) while targeting a cardiovascular intensity of 70–85% of HRmax and RPEs of 14–17 (nearly hard to very hard). The formula 211 − (0.64 × age) was used to calculate HRmax, and it was selected because it was developed on data from a Norwegian cohort [24]. The HRmax was reduced by 15 bpm if the participant used beta-blockers [25,26,27]. Heart rate was monitored continuously during PT sessions using OH1 (Polar Electro, Kempele, Finland) armbands, which provided real-time feedback on the participants’ HR to the clinicians. HR responses to the different activities guided clinical decision-making to maximize time in the target heart rate zone. In accordance with the protocol for HIT, the aims of the sessions were to spend 50% of the active time walking on a treadmill, 25% walking overground, and 25% on stairs [19]. However, the physical therapists were allowed to prioritize activities depending on HR response and the participant’s individual needs and preferences. Transitions between tasks were accompanied by a short rest break if needed (1–2 min). Aside from this, rest breaks were not encouraged by the physical therapists, but were given when requested by the participant.

Blood pressure was monitored before, during, and after each PT session. Local guidelines were set by the supervising physician, with participants precluded from exercising when systolic blood pressure was outside 100–180 mmHg or when diastolic blood pressure was above 110 mmHg. As recommended in previous COVID-19 studies [28,29], the protocol was additionally modified to include the monitoring of oxygen saturation (SpO_2_) during PT assessments and training interventions. Physician approval was required to continue the PT session if SpO_2_ fell below 88% [30].

Outside of scheduled PT sessions, participants performed pulmonary rehabilitation techniques as part of a self-training program. This included diaphragmatic breathing, inspiratory hold and stacked breathing, and inspiratory muscle training (IMT) using an IMT device (Threshold IMT, Philips, Amsterdam, Netherlands). The resistance provided by the IMT device was set to 50% of the participant’s most recent maximal inspiratory pressure (MIP), which was reassessed weekly [31]. The participants were encouraged to perform 5 sets of 6 breaths with 30–60 s of rest between sets, two times per day. Other concurrent interventions included occupational therapy and speech and language pathology when indicated. The nurses also assisted with activities of daily living and walking practice as a component of the interdisciplinary care provided during inpatient rehabilitation.

### 2.3. Data Collection

The project was approved by the Regional Ethics Committee South-East, Norway [23 March 2021/154279]. Data were collected and stored per the protocol and guidelines of the Norwegian Centre for Research Data.

#### 2.3.1. Demographics and Health Records

Demographic data, including age, sex, body mass index (BMI), smoking history, comorbidities, time since initial positive COVID-19 test, length of stay, and COVID-19-related complications during the acute hospitalization were collected from the patient’s medical records.

#### 2.3.2. Feasibility and Safety of the Intervention

Physical therapists documented vital signs including heart rate, RPE, SpO_2_, blood pressure, and interventions provided during PT sessions. Stepping activity was collected during PT sessions and throughout the day using the StepWatch 4 (Modus Health, Edmonds, WA, USA). During PT sessions, OH1 armbands measured the cardiovascular intensity of the training. RPEs were measured on the 6–20 point Borg Scale. Blood pressure was monitored before, during, and after training using manual (Minimus^®^ III Sphygmomanometer, Riester, Jungingen, Germany, and a Littmann^®^ Lightweight II S.E. stethoscope, 3M™, Maplewood, MN, USA) or automatic (ProBP 3400, Welch Allyn, Skaneateles Falls, NY, USA or CARESCAPE V100, GE HealthCare technologies Inc., Chicago, IL, USA) equipment. SpO_2_ was monitored for each task during PT sessions using a pulse oximeter with a finger sensor (RAD-5 or the MightySat Rx, Masimo Corp., Irvine, CA, USA). The feasibility of HIT was determined by the participants’ steps and the HRs achieved in the sessions. If feasible, the participants should be able to achieve similar or greater results than in Moore et al. (2020). Specifically, participants of that study achieved an average of 1866 (±653) steps per PT session and spent over 30% of the session time at cardiovascular intensities >70% of HR max [23].

Adverse events during and outside of PT sessions were manually documented by the unit’s staff and checked against the participant’s medical chart for completeness. The severity of adverse events was graded on a 1–5 scale by one experienced physical therapist according to the Common Terminology Criteria for Adverse Events (CTCAE, v5) [32]. Grade 1 adverse events indicate mild symptoms or signs with no intervention indicated whereas those graded 2–5 indicate the patient needs intervention, with classifications of “moderate”, “severe”, “life-threatening”, and “death”, respectively.

#### 2.3.3. Functional Outcome Measures

The participants were assessed with a standardized assessment battery within one week of admission and discharge. While standardized assessments have not been psychometrically tested in patients with COVID-19, their utility and psychometric properties in pulmonary and other relevant conditions informed their selection. In addition, some measures were recommended for use in rehabilitation for individuals recovering from COVID-19 by the South-Eastern Norway Regional Health Authority [33].

All standardized assessments were administered by experienced physical therapists. Training for the raters included semiannual workshops that focused on standardized administration procedures and theoretical and practical reviews of the administration protocols.

The ability to perform activities of daily living was assessed using the Barthel Index (0–20 point version). Gait speed was collected using the 10 m walk test (10MWT) at both self-selected speed (SSS) and fast speed (FS) [34]. Walking capacity was determined using the 6 min walk test (6MWT) [35,36], while walking independence was assessed using the functional ambulation categories (FAC) [37].

Balance was assessed using the Berg balance scale (BBS), the mini-balance evaluation systems test (Mini-BESTest), and the short physical performance battery (SPPB). The BBS is a standardized postural stability and balance assessment commonly used in rehabilitation [38,39]. The Mini-BESTest assesses anticipatory postural adjustments, reactive postural responses, sensory orientation, and dynamic gait [39]. The SPPB consists of three functional items that include static balance, walking function, and five sit-to-stands [40,41,42].

Respiratory muscle function was assessed by testing the MIP and maximal expiratory pressure (MEP). Tests were performed with the MicroRPM Respiratory Pressure Meter (Micro Direct Inc., Lewiston, ME, USA) and reported in cm H_2_O.

### 2.4. Statistical Analysis

Analyses were performed in SPSS Statistics V27 (Release 27.0.1.0, IBM, Armonk, NY, USA). The data were tested for normality using the Shapiro–Wilk test. Categorical data are presented as n (%), while continuous data are presented as mean (±SD) or median (Q1–Q3), depending on whether they were normally distributed. Descriptive analyses were performed on the types of interventions provided during PT sessions. HIT interventions were stratified by their training environment (i.e., treadmill, overground, stairs) with their relative distribution reported.

Descriptive analyses were performed for stepping activity during the inpatient stay. In addition to steps/day, the total number of steps, stepping rate, and minutes of stepping activity were extracted from each PT training session. The cardiovascular intensity during PT sessions was reported as the peak HR achieved, and the percentage of the PT session time spent at ≥70% HRmax. Comparisons between admission and discharge were performed using paired t-tests, reported in mean (±SD), and Wilcoxon’s signed-rank tests, reported in median (Q1–Q3), depending on whether the data met the assumption of parametric tests. The alpha was set at 0.05 and the analyses were performed with Bonferroni-adjusted *p*-values to account for multiple comparisons.

## 3. Results

### 3.1. Sample and Demographics

Between 1 April 2021, and 28 February 2022, 34 patients post-COVID-19 were admitted to inpatient rehabilitation. Of these, 22 (64.7%) patients met the inclusion criteria and consented to participate in the pilot study, while 8 also consented but were not medically cleared for HIT. Four patients were unable to consent or had bracing or other instrumentation that substantially restricted movement. Of the 22 participants medically cleared for HIT, two later withdrew their consent, leaving data from 20 participants available for analysis. Demographic data are shown in Table 1.

### 3.2. Fidelity of the Intervention

The participants received a median of 3.4 (3.0–3.9) PT sessions per week that lasted 53.3 (48.1–56.4) minutes. Throughout their inpatient stays, the participants received a median of 10.0 (7.3–17.3) PT sessions in total.

Daily treatment documentation indicated HIT was provided in all PT training sessions and only a single session 1/301 (0.3%) included a non-walking intervention (strength training). While providing HIT, 40.0% (8.0–58.9) of the time was spent walking on a treadmill, 29.0% (22.0–73.9) on walking overground, and 16.7% (9.2–30.2) on stairs.

Participants took an average of 4813 (±3061) steps/day. During PT sessions, participants performed an average of 2093 (±619) steps/session, by walking for 35.1 (±6.3) minutes at a rate of 55.1 (±8.8) steps/minute. HR data were unavailable for three participants; however, for the remaining participants, the average peak heart rate during the PT sessions was 81.1% (±9.4) HRmax, and 30.1% (±21.0) of the session time was spent at heart rates of ≥70% HRmax. The peak RPE was 16.8 (15.1–18.0), indicating the participants were working very hard. During PT sessions, the average drop in SpO_2_ was 6.2 (±3.3) percentage points with the lowest SpO_2_ exhibited during training, averaging 87.9% (±4.9). In 42.5% (0.0–80.8) of the PT sessions, supplemental oxygen was used.

### 3.3. Safety of the Intervention

Exercise-related grade 1 adverse events were reported in eleven participants (55%), but PT sessions in which events were reported (*n* = 15) accounted for <5% of the total number of sessions. Adverse events reported during PT sessions include musculoskeletal pain (*n* = 5), dizziness (*n* = 3), nausea (*n* = 3), hypotension (*n* = 3), hypertension (*n* = 1), and transient chest pain (*n* = 1). In addition, non-exercise-related grade 1 adverse events reported before initiating the PT sessions were nausea (*n* = 3), tachycardia at rest (*n* = 1), transient chest pain (*n* = 1), infection (*n* = 1), dizziness (*n* = 1), and low blood sugar (*n* = 1). One patient experienced two falls outside PT, causing musculoskeletal pain but no significant injuries. None of the participants experienced any grade 2–5 adverse events or were readmitted to a higher level of care during their inpatient rehabilitation stay.

### 3.4. Functional Outcome Measures

Results from the standardized assessments at admission and discharge are shown in Table 2. Normative values for an age-matched population are also included when available. Importantly, 85% (17/20) were discharged home with the remaining patients being discharged to other specialized rehabilitation units.

## 4. Discussion

This pilot study provides the first evidence detailing the feasibility and safety of HIT in individuals undergoing inpatient rehabilitation post-COVID-19. Included participants were typically older adults and most had comorbidities and required treatment with supplemental oxygen and a ventilator during acute hospitalization, similar to other published trials of participants undergoing post-COVID-19 inpatient rehabilitation [48,49,50,51], with the functional level at admission being similar [49] or somewhat higher [50,51] in our sample.

The results from this pilot study show that the PT sessions were almost exclusively comprised of task-specific walking performed on the treadmill, overground, or stairs. Compared to the HIT training protocol by Holleran et al. (2014), where the treadmill was used 50% of the active time, overground walking comprised 25%, and use of stairs 25% [19], our sample spent slightly more time training overground and less time on the treadmill and stairs. However, the stepping metrics indicate that the participants were still achieving a high number of steps, both during and outside the PT sessions. Therefore, since the steps per PT session were higher than previously reported in Moore et al. (2020), it was feasible to obtain high stepping activity in this population [23]. To the best of our knowledge, no previous studies have reported stepping activity during inpatient rehabilitation post-COVID-19. De Souza et al. (2021) reported steps per day before (8671 ± 1355) and after (10,492 ± 1122) a low-intensity pulmonary rehabilitation program post-COVID-19 [52]. However, this study was performed with participants who did not require intensive care and who had significantly higher functional levels at baseline.

On average, participants in this pilot study demonstrated peak heart rates above 80% HRmax and spent just over 30% of the PT session at heart rates ≥ 70% HRmax. These data indicate that achieving higher cardiovascular intensities with this intervention was feasible in individuals post-COVID-19. Mohamed and Alawna (2021) previously investigated moderate-intensity training (60–75% HRmax) post-COVID-19; however, the fidelity of the intervention (i.e., heart rates achieved) was not reported [15]. In addition, the calculations used for predicted HRmax (HRmax = 210 − age) differed from this pilot study (HRmax = 211 − 0.64 × age) and results in lower estimations of HRmax as it has a higher subtraction for the participant’s age, and correspondingly results in lower target heart rate zones. Corna et al. (2022) also used a different formula (HRmax = 220 − age) when calculating the predicted HRmax, resulting in lower predictions of HRmax and a lower target heart rate zone for individuals >25 years of age. Their results indicate that the participants achieved HRs between 60–70% of HRmax which was the lower end of the targeted intensities of 55–85% of predicted HRmax [14]. Mooren et al. (2023) did not report target intensity or fidelity using HR [16]. However, they did report a mean exercise HR of 113.9 ± 14.4 in the aerobic interval group and 111.1 ± 15.0 in the continuous training group. If using their reports of mean age to calculate predicted HRmax with the same formula used in this pilot study, their results indicate participants in the aerobic and continuous training groups had an average exercise HR of 63.5% and 61.7% of predicted HRmax, respectively. Results from the study by Foged et al. (2021) indicate that the participants reached intensities > 85% HRmax during the three high-intensity interval protocols investigated [17]. However, the participants in their study were only mildly affected by persisting COVID-19 symptoms and may not be comparable to the present sample. Similarly, 12 of the 14 participants in the high-intensity interval training group in the study by Rasmussen et al. (2023) achieved the fidelity criteria (≥25% of the training time with HR > 85% of HRmax) [18]. However, the study did not include participants who required intensive care during the COVID-19 infection, and the results might not be generalizable to the population commonly seen during inpatient rehabilitation. Our results reinforce the existing findings that HIT is feasible post-COVID-19 and provide preliminary evidence that it is feasible during routine inpatient rehabilitation.

In terms of the safety of the intervention, mild adverse events (grade 1) occurred in <5% of the 301 PT sessions provided in this pilot study, and no intervention-requiring or life-threatening adverse events (grade 2–5) occurred. Limited data are available to describe adverse events resulting from specifically high-intensity protocols and exercise in general for patients undergoing rehabilitation post-COVID-19. Neither Mohamed and Alawna (2021) nor Mooren et al. (2023) reported adverse events [15,16]. Rasmussen et al. (2023) reported 11 adverse events in the experimental group, including angina (*n* = 1), infection/illness (*n* = 3), hypotension (*n* = 1), hypertension (*n* = 1), physical complaints (*n* = 4), and fall (*n* = 1), which were all classified as not related to the intervention [18]. However, they defined adverse events as symptoms lasting > 24 h or hospitalization ≤ 24 h, and serious adverse events were classified as hospitalization > 24 h. As transient symptoms during training or symptoms only persisting ≤ 24 h were not reported, it is difficult to compare their results to this pilot study. Although Foged et al. (2021) reported that no adverse events occurred during three high-intensity interval training protocols, only 30 sessions were performed during the study [17]. In addition, the authors did not list the adverse events that were monitored, thus making it difficult to compare as minor adverse events might not have been reported. Corna et al. (2022) reported no serious adverse events, and only a few mild adverse events. However, reported symptoms during sessions included fatigue (*n* = 6), and muscular pain and dyspnea (*n* = 4). Also, two participants missed seven sessions due to blood pressure- or HR-related issues. In total, 17 mild adverse events occurred during the 160 sessions of aerobic training, resulting in a calculated incidence of just over 10% [14].

In studies assessing other exercise interventions for patients post-COVID-19, some adverse events have been reported. In a RCT investigating a home-based moderate intensity aerobic exercise and strengthening program, 42.4% of the participants in the intervention group experienced mild adverse events including chest tightness (22.2%), dizziness (13.3%), and chest pain (11.1%) during the intervention period [53]. Dizziness and transient chest pain were also observed in our sample, but with a higher number of blood pressure-related events (25%), which was not specifically reported in the study by Li and colleagues. However, the total number of events is comparable to our pilot study, where 55% of the participants experienced a mild exercise-related adverse event during the PT sessions. A large systematic review by Niemeijer et al. (2020) found that participating in exercise interventions led to an increase in the relative risk of non-serious but not in life-threatening adverse events across varied patient populations [54]. Importantly, no life-threatening adverse events occurred in this pilot study of HIT during inpatient rehabilitation post-COVID-19.

As shown in Table 2, the sample in this pilot study demonstrated impaired walking function, balance, and respiratory muscle function at admission as compared to normative values for an age-matched sample [40,43,44,45,46,47]. Walking speed can predict discharge location after rehabilitation, the ability to ambulate in the community, and general health status [55]. Improved walking function and mobility are also among the most reported patient goals in subacute rehabilitation across different populations [56,57]. Therefore, improving walking function during inpatient rehabilitation is often a priority. Focusing on gait activities such as overground and stair walking is a task-specific strategy to improve walking function, including gait speed and distance. HIT has been previously found to substantially improve walking function in other diagnoses, especially following neurologic injury [19,20,21,22,23]. In this sample, gait-related outcomes significantly improved between admission and discharge, which, although not specifically validated post-COVID-19, likely indicated a positive impact on the patients’ walking function. Additionally, although balance may not have been a primary deficit for the participants in this pilot study, previous studies have identified reduced balance function in individuals post-COVID-19 compared to both individuals with an acute exacerbation of chronic obstructive pulmonary disease [58] and healthy subjects [58,59], indicating that this is an important impairment to address. In this pilot study, improvements were seen in all balance measurements at discharge and were aligned with normative values. Finally, respiratory muscle function was substantially lower than normative values at admission but approached normative values at discharge. Optimizing function in the subacute stage post-COVID-19 could have implications for long-term symptoms and quality of life for these individuals.

There are several limitations to this pilot study. First, the small sample was admitted to inpatient rehabilitation with a relatively high functional level at admission, which should be considered regarding the generalizability of the results. Second, the study design does not allow for the investigation of the comparative effectiveness of HIT versus other interventions for this population regarding short- and long-term outcomes, as it does not include a control group. It is therefore not known whether the improvements in outcomes are due to HIT, the other interventions provided during inpatient rehabilitation or due to spontaneous recovery. Additionally, PT session activities and adverse events were manually documented by the physical therapists, and some activities or events may have been missed. There might also be variations in the subjectively reported symptoms during exercise due to the participant’s willingness to share. Furthermore, since this pilot study occurred during an inpatient rehabilitation stay, other interventions were provided by nursing and occupational therapists and could have influenced observed changes in outcomes. The participants were also instructed in breathing techniques and IMT as self-training, but the adherence to these interventions was not monitored and their potential impact on functional outcomes are unclear. Finally, although potentially important demographical factors (smoking history, CCI, LOS, etc.) were collected, their influence on the feasibility and safety of HIT, or the rehabilitation process in general, were not investigated in this pilot study. Future research should consider the influence of these factors on inpatient rehabilitation post-COVID-19.

## 5. Conclusions

This pilot study provides preliminary evidence that exercise at 70–85% of predicted HRmax may be feasible and safe during inpatient rehabilitation for medically cleared patients post-COVID-19. Future studies should investigate the comparative effectiveness to determine whether this intervention results in superior outcomes than other exercise interventions. As a large portion of individuals surviving COVID-19 report lasting symptoms with reduced function and quality of life, investigating the long-term benefits of the intervention would also be of importance.

## Figures and Tables

**Table 1:** Demographics and COVID-19-related complications during the acute hospitalization.

Description	Result
Age, years (*n* = 20)	62.35 (±14.02)
Sex, female (*n* = 20)	11 (55%)
Body mass index (BMI), kg/m^2^ (*n* = 12)	27.10 (±3.90)
Smoking history, never/previous/active (*n* = 18)	6 (33%)/11 (61%)/1 (6%)
Time since COVID-19 infection, days (*n* = 19)	58.89 (±26.85)
LOS at unit, days (*n* = 20)	27.50 (18.50–34.75)
- Charlson comorbidity index (CCI)	2.95 (±1.73)
Complications in the acute stage (*n* = 20)	
- Post-intensive care syndrome	1 (5%)
- Critical illness myopathy	2 (10%)
- Critical illness polyneuropathy	1 (5%)
- Chronic inflammatory demyelinating polyneuropathy	1 (5%)
- Pulmonary embolism	1 (5%)
Mechanical ventilator in hospital, yes (*n* = 20)	15 (75%)
Days on mechanical ventilator, days (*n* = 12)	29.7 (±18.7)
Oxygen treatment in hospital, yes (*n* = 15)	14 (93%)

**Table 2:** Functional outcomes at admission and discharge, change score between admission and discharge, and statistical significance of change. Note that normative values describe individuals aged 60–69 years except MEP, which apply to individuals aged 65–69 years.

Description	Admission	Discharge	Change	*p*-Value	Normative Data
Barthel Index (*n* = 18)	16.0 (11.8–18.3)	20.0 (18.8–20.0)	3.5 (1.0–7.0)	<0.001	-
10 MWT SS, m/s (*n* = 20)	0.78 ± 0.31	1.11 ± 0.26	0.33 ± 0.21	<0.001	1.24–1.34 [43]
10 MWT FS, m/s (*n* = 19)	1.17 ± 0.47	1.53 ± 0.36	0.36 ± 0.37	<0.001	1.87–2.05 [44]
6 MWT, m (*n* = 19)	259.8 ± 128.0	400.4 ± 108.5	140.6 ± 90.5	<0.001	538–572 [44]
FAC (*n* = 20)	4.0 (4.0–5.0)	5.0 (4.0–5.0)	1.0 (0.3–1.8)	<0.001	-
BBS (*n* = 20)	49.0 (20.8–53.0)	55.0 (47.0–56.0)	4.5 (1.3–20.5)	<0.001	55 [44]
MiniBESTest (*n* = 19)	15.0 (7.0–23.0)	24.0 (19.0–25.0)	5.0 (2.0–12.0)	0.001	24.7 [45]
SPPB (*n* = 19)	7.0 (3.0–10.0)	11.0 (6.0–12.0)	2.0 (0.0–5.0)	0.002	11.4–11.7 [40]
MIP, cm H_2_O (*n* = 20)	59.4 ± 28.5	77.7 ± 28.9	18.3 ± 17.2	<0.001	75.1–92.7 [46]
MEP, cm H_2_O (*n* = 20)	59.5 (50.3–95.0)	105.0 (73.5–112.0)	15.5 (0.3–39.5)	<0.001	125.0–188.0 [47]

## Data Availability

The data presented in this study are available on reasonable request from the corresponding author. The data are not publicly available due to local policies.
